# Selection of best videos of the year for 2023

**DOI:** 10.1590/S1677-5538.IBJU.2024.01.03

**Published:** 2024-03-18

**Affiliations:** 1 Department of GU Oncology Moffitt Cancer Center FL United States Department of GU Oncology Moffitt Cancer Center, FL, United States; 2 Department of Tumor Biology Moffitt Cancer Center FL United States Department of Tumor Biology Moffitt Cancer Center, FL, United States

Dear readers,

I very much hope this message finds you well. Firstly, I wanted to thank each and everyone of you for your support and commitment to our journal over the recent years. The International Brazilian Journal of Urology has had a dramatic rise in its impact factor as many of you may be aware (currently IF of 3.70 for 2023), with much of this recognition deserved by the editor-in-chief Professor Luciano A. Favorito and the entire editorial board and notably the commitment of our readers. Many of you as authors have selected our journal for the submission of your quality work including as innovative and beautifully depicted video submissions. In this regard, one of my greatest joys and honors every year is to highlight the selection of the three best videos of the year within the International Brazilian Journal of Urology. This selection for best videos of the year is incredibly challenging as most of the videos published (and often submitted to us) are of high quality in terms of quality, novelty, and potential to redefine the current treatment paradigm. Despite these inherent challenges, I am pleased to share with all of you my selection for best videos of the year. The 1st prize is awarded to the video by Santos et al. from the AC Camargo Cancer Center in Brazil entitled “Primary laparoscopic RPLND for pure seminoma metastasis: feasibility of supine and lateral approaches” published in volume 49, number 2 of this year's published issues (1). As is very well established in the scientific literature for the surgical management of retroperitoneal disease disseminating from testicular seminoma, these operative cases can be challenging although many traditionally have been conducted in the post-chemotherapy setting. With the recent conduct and publication of two recent trials (SEMS and PRIMETEST), an evolution in the surgical contemplation for low volume (clinical stage IIA and select IIB) retroperitoneal disease consisting of seminoma is being investigated with this video and abstract highlighting how it can be completed in a minimally invasive manner by highly skilled surgeons as is depicted here. The authors share their tips and tricks in accomplishing such procedures, with favorable outcomes. They are to be congratulated on their significant contribution to the field, understanding that such cases should only be completed in the setting of a clinical trial and by highly skilled minimally invasive surgeons, with a low threshold for open conversion if adequacy of surgical resection is at all felt to be compromised. The second prize for best video of the year is awarded to Nunes, et al. from the University of São Paulo for their submission entitled “Laparoscopic ureterocalicostomy technique” published in volume 49, number 3 of our journal (2). As we all know, reconstruction using an ureterocalicostomy is infrequently completed by even experienced urologists so this publication using a minimally invasive approach and marvelously captured and described represents an invaluable resource for urologists and trainees. I would encourage our readers to share this resource with their colleagues as it highlights how this reconstructive technique can be used to address challenging proximal ureteral defects/resections, ideally in the setting of a dilated renal pelvis/upper urinary system. The third prize for best videos of the year is awarded to Sandberg, et al. from Wake Forest University in North Carolina (USA) for their submission entitled “Robotic assisted radical nephrectomy with inferior vena cava tumor thrombus” published in volume 49, number 5 of our journal (3). Although the minimally invasive approach to complex urologic oncology cases such as kidney cancer with intravascular venous thrombus has been well reported in the recent peer reviewed scientific literature, the present video details very elegantly how even high level IVC thrombi can be tackled using meticulous technique and adherence to key principles of vascular surgery including proximal/distal control, lumbar vessel ligation, and ensuring the entire tumor thrombi is resected (understanding that if vascular wall invasion is in fact seen at time of surgery it will necessitate resection and possible vascular patching, grafting, or in certain select circumstances ligation (if collateral flow can be established over time).

Lastly, I would like to take this opportunity to thank all our authors readers. Your support and commitment really assures, the continued success of our journal and we look forward to continuing to highlight innovation and surgical enhancement in trying to improve the care we deliver to our patients.

Very best wishes to all of you and your families for the holidays and New Year.

Very much hope to see you all soon!

Warm regards and best wishes
